# Towards scalable nano-engineering of graphene

**DOI:** 10.1038/srep07314

**Published:** 2014-12-04

**Authors:** A. J. Martínez-Galera, I. Brihuega, A. Gutiérrez-Rubio, T. Stauber, J. M. Gómez-Rodríguez

**Affiliations:** 1 Departamento Física de la Materia Condensada, Universidad Autónoma de Madrid, E-28049 Madrid, Spain; 2 Condensed Matter Physics Center (IFIMAC), Universidad Autónoma de Madrid, E-28049 Madrid, Spain; 3 Instituto de Ciencia de Materiales de Madrid, Consejo Superior de Investigaciones Científicas, E-28049 Madrid, Spain

## Abstract

By merging bottom-up and top-down strategies we tailor graphene's electronic properties within nanometer accuracy, which opens up the possibility to design optical and plasmonic circuitries at will. In a first step, graphene electronic properties are macroscopically modified exploiting the periodic potential generated by the self assembly of metal cluster superlattices on a graphene/Ir(111) surface. We then demonstrate that individual metal clusters can be selectively removed by a STM tip with perfect reproducibility and that the structures so created are stable even at room temperature. This enables one to nanopattern circuits down to the 2.5 nm only limited by the periodicity of the Moiré-pattern, i.e., by the distance between neighbouring clusters, and different electronic and optical properties should prevail in the covered and uncovered regions. The method can be carried out on micro-meter-sized regions with clusters of different materials permitting to tune the strength of the periodic potential.

Two main routes are usually followed to modify graphene's electronic and optical properties. On the one hand, bottom up approaches have proven to be efficient to change the overall electronic structure of graphene, enabling for example, the gap opening at the Fermi energy^1^,^2^,^3^, renormalization of the Fermi velocity^4^,^5^,^6^ or controllable n- and p-type electronic doping^7^,^8^,^9^,^10^. On the other hand, with top down approaches it is possible to induce these alterations on a local scale enabling one to pattern graphene to quantum confine electrons^11^,^12^,^13^,^14^, to induce local magnetic and superconducting properties^15^,^16^, or to use a scanning probe to selectively tune its electronic properties^13^,^17^,^18^. Still, a remaining challenge is the realization of controlled nanopatterning below 10 nm sizes^19^,^20^, key for the comprehensive integration of graphene in real devices. Here, we show that combining both approaches, i.e., bottom-up and top-down, one can reach a 2.5 nm patterning, enriching graphene's capabilities even more.

Let us first outline the bottom-up approach^1^,^2^,^4^,^5^,^21^,^22^ for graphene monolayers on several metallic substrates which can be epitaxially grown with unrivaled quality^23^. An interesting common feature of most of these graphene-metal interfaces is the presence of superperiodicities, known as Moiré patterns, resulting from the lattice mismatch and rotation angle between graphene and metal lattices^24^. This creates a periodic potential superimposed to graphene whose strength can be tuned by the preferential adsorption of different adsorbates on specific positions of the Moiré superlattice^1^,^2^,^3^,^25^. A graphene metal interface particularly interesting for our purposes is the graphene monolayer epitaxially grown on Ir(111) substrates. It allows for growing single Moiré domains extending over micrometers^25^,^26^ while at the same time, the interaction with the substrate remains weak leaving almost unaltered the electronic properties of the graphene layer, i.e., the *π*-bands with the characteristic linear dispersion and Fermi velocity of free standing graphene are only modified by the appearance of a small gap less than 100 meV^2^,^27^,^28^, see Fig. 1b.

Additionally, this Moiré pattern formed by the graphene monolayer and the Ir(111) substrate can be used as a template for networks of monodisperse clusters of transition metals^25^,^29^. As recently reported, the adsorption of these cluster superlattices strengthens the periodic potential created by the Moiré pattern, modifying the electronic properties of the graphene layer^2^. In particular, an increase of the band gap up to 400 meV and large anisotropies of the electron group velocity close to the Dirac point have been measured for Ir cluster superlattices^2^, see Fig 1c.

## Results

The experimental bottom-up procedure is the following. We first grow a graphene monolayer on an Ir(111) substrate by chemical vapor deposition (CVD) of ethylene in UHV environments with the Ir(111) substrate held at 1050°C. Then, by evaporationg W or Ir from high purity filaments, we subsequently cover it with a hexagonal array of metal clusters with 2.5 nm periodicity (see methods for details on the sample preparation).

**Figure 1:**
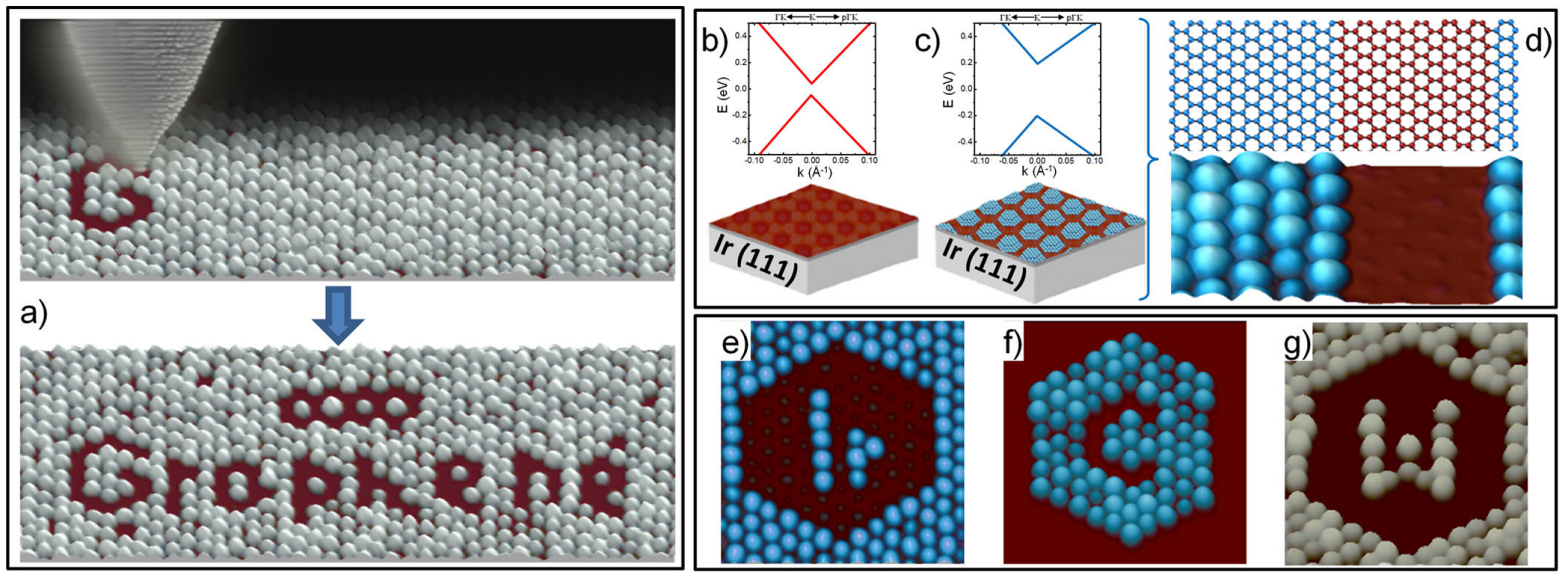
Tailoring graphene with 2.5 nm accuracy. (a) Upper panel illustrates the patterning process, with a schematic STM tip drawn on top of a real experimental image, removing selected W clusters from the G/Ir(111) surface to write the word “graphene”. Lower panel shows a 95 × 35 nm^2^ STM image with the final result. (b), (c) Graphene π bands, in the vicinity of E_F_, for pristine G/Ir(111) and G/Ir(111) covered with an Ir cluster superlattice respectively, as measured by photoemission in ref (2). (d) Example of a graphene-based nanostructure formed by two different “graphene” with the electronic properties depicted in (b) and (c). (e) – (g) 30 × 30 nm^2^ STM images showing the validity of our method for clusters of different materials, in particular Ir (e,f) and W (g). Tunneling parameters: I_T_ = 20 pA, V_s_ = +2.2 V (a); I_T_ = 150 pA, V_s_ = +1.5 V (d); I_T_ = 150 pA, V_s_ = +1.5 V(e); I_T_ = 160 pA, V_s_ = +2.0 V (f); I_T_ = 40 pA, V_s_ = +1.5 V (g). We have used the following color code in all our images: reddish corresponds to pristine G/Ir(111), bluish to Ir clusters and grayish to W ones. All STM data were acquired and analyzed using the WSXM software^46^.

We will now turn to the above mentioned top-down approach where STM appears as an ideal technique to tackle local manipulation on such samples due to its ability to modify and pattern 2D samples with ultimate resolution^30^,^31^,^32^. In Fig 1a, we write “graphene” on the G/Ir(111) surface using the STM tip to completely remove the selected clusters which demonstrates the patterning on top of graphene with 2.5 nm accuracy and a very high degree of complexity, see also examples of Figs 1 e–g. In this way, by deliberately removing metallic clusters from the graphene layer, we can recover the electronic properties corresponding to the pristine G/Ir(111) interface in specific regions of the sample and can architecture nanostructures formed by two different kinds of *‘graphene’* regions, i.e., ones covered with clusters and uncovered ones, with supposedly different electronic properties. It is noteworthy that our method can be used with clusters of different metallic elements, see Figs 1e–g where Ir (e–f) and W (g) clusters formed by approx. 50 atoms have been removed. This might allow tuning the strength of the periodic potential superimposed to the graphene layer and, consequently, the electronic and optical properties for the covered regions.

**Figure 2:**
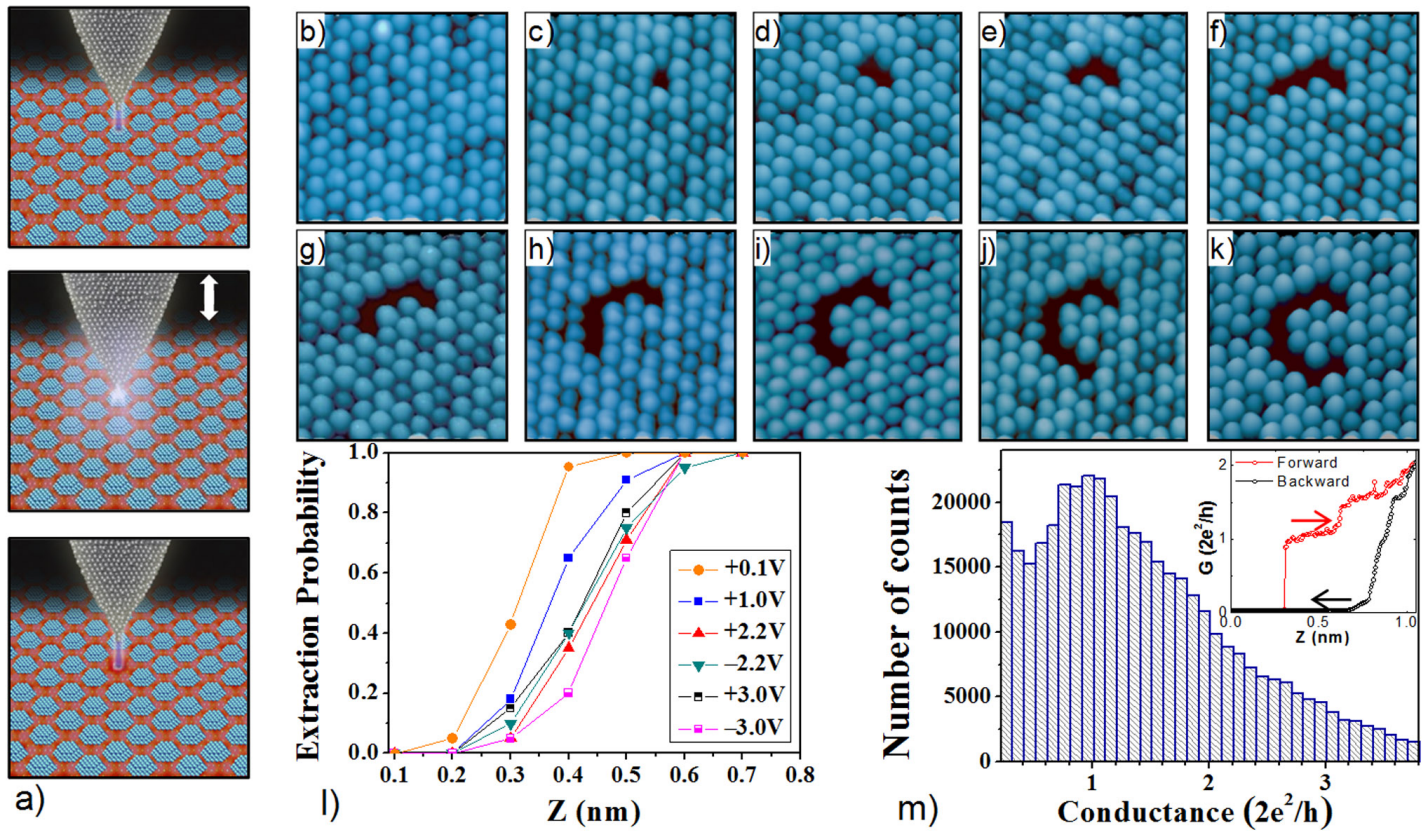
Cluster extraction procedure. (a) Illustration of our cluster extraction method by the vertical displacement of a STM tip. (b–k) Sequence of 23 × 23 nm^2^ STM images, showing the writing of the carbon chemical symbol by consecutively removing one by one Ir clusters. All the images were acquired at RT with I_T_ = 160 pA and V_s_ = +2.0 V. To remove each cluster, the STM tip was approached 0.6 nm to the surface at 100 mV. (l) Curves of the probability of removing a cluster as a function of approaching distance for several applied voltages. In all cases, stabilization current was set to 70 pA before opening the feedback loop. (m) Conductance histogram constructed from I(z) measurements on 1200 single cluster extraction events. Each curve was obtained at RT, with 0.1 V sample voltage. Inset shows an example of the conductance curve recorded during one of such cluster extraction events.

The procedure we have developed to engineer graphene nanostuctures consists in selectively removing single metallic clusters on top of graphene by gently approaching the STM tip towards them, as schematized in Fig 2a. We first image a large graphene sample area completely covered with metallic clusters, see Fig 2b. Next, we choose a metal cluster to be removed and stop the STM tip above it. With the tip above the chosen cluster, we open the feedback loop and bring the tip towards the sample at a constant rate for a distance of typically 0.6 nm. Then, we retract the tip back and close the feedback loop returning to the initial tunneling conditions. This completely removes the selected cluster as shown in Fig 2c. Finally, we systematically repeat this procedure to remove all selected clusters and thus form the designed nanostructure. As an example, the complete sequence for writing a “C” by consecutively removing 9 Ir clusters is shown in Figs 2b–k.

During the patterning process, the tip resolution is very robust and we usually observe almost no changes in our resolution after each cluster removal (see supplementary material). It further appears that the extracted clusters wet the STM tip and indeed, we remove the metal cluster as a whole since no traces of metal atoms are observed on the graphene surface after each single extraction event. This is likely due to the large cohesive energy of both W and Ir compared to the binding energies of C-Ir and C-W, respectively^25^,^33^. Such high cohesive energies together with the strong W-Ir binding should thus be responsible of the observed tip stability; once the cluster material wets the STM tip, it remains there in an extremely stable manner such that we have not been able to place the metal cluster back on the graphene surface.

The possibility of picking up or manipulating individual clusters formed on the Moiré-pattern was previously mentioned^25^,^34^. But on these works the cluster manipulation was a rather rare and statistical event. In fact, it was even considered a disturbing effect since it happened more or less statistically during the scanning process that could only be avoided under suitable tunneling conditions. Our work thus goes far beyond these earlier observations as we are now able to demonstrate that these cluster manipulations can be controlled to form arbitrary patterns stable even at room temperature. An essential issue regarding the validity of the procedure just described thus stems from its actual efficiency to extract the selected clusters.

To this end, we have performed a careful study of the probability of removing a cluster as a function of both the tip-sample approaching distance and the bias voltage applied to the sample during the whole process, see Fig. 2l and the supplementary material for details. The most important finding is that, for all voltages investigated during this study, we can reach a 100% probability for extracting a cluster by approaching the tip towards the sample a distance exceeding a certain value, between 0.5–0.7 nm, slightly different for each voltage. This allows to nanopattern the graphene surface with almost any degree of complexity and perfection. We also observed that, while the probability of extracting a cluster strongly depends on the approaching distance, the dependence on the applied voltage is much more moderate and basically independent of the voltage polarity, i.e., the direction of the electric field between tip and sample. For all voltages investigated here, the shape of the probability curves is essentially the same with only a rigid shift between them. This shift originates from the initial tip-sample distance dependence on the bias voltage set prior to open the feedback loop. Thus, our results point to a cluster removal procedure mainly driven by the actual distance between the STM tip and metal cluster.

To get more insight into the physical processes involved in the cluster extraction, we recorded the current during the vertical displacement of the STM tip (I-Z curves), see for example inset of Fig 2m. As usual when investigating the approach between two metallic electrodes, individual conductance curves were inherently irreproducible (see supplementary material), which is generally attributed to variations in the actual atomic-scale configuration of the metallic electrodes during the transition from tunneling to direct contact^35^,^36^,^37^,^38^. Thus, to perform an objective analysis of our experimental data, we constructed a conductance histogram from the evolution of the conductance traces of more than a thousand single cluster extraction events, see Fig 2m. Peaks in such conductance histograms are related to statistically more probable configurations in the contact formation^36^,^37^,^38^. The histogram shows a clear peak for a quantum of conductance (*G_0_* = 2*e*^2^/*h, e:* electron charge*; h:* Planck's constant), indicating that the extraction of a metal cluster involves the formation of an atomic size contact. Similar *G* ≈ *G_0_* values have been reported for contacts between an atomically sharp Au tip and graphene regions strongly bonded with a metal substrate^39^. In such regions, carbon atoms bind strongly to the metal surface and the hybridization of the graphene orbitals is transformed from sp^2^ to sp^3^, in a similar way as reported for graphene regions underneath metal clusters on the graphene/Ir(111) system^33^.

**Figure 3:**
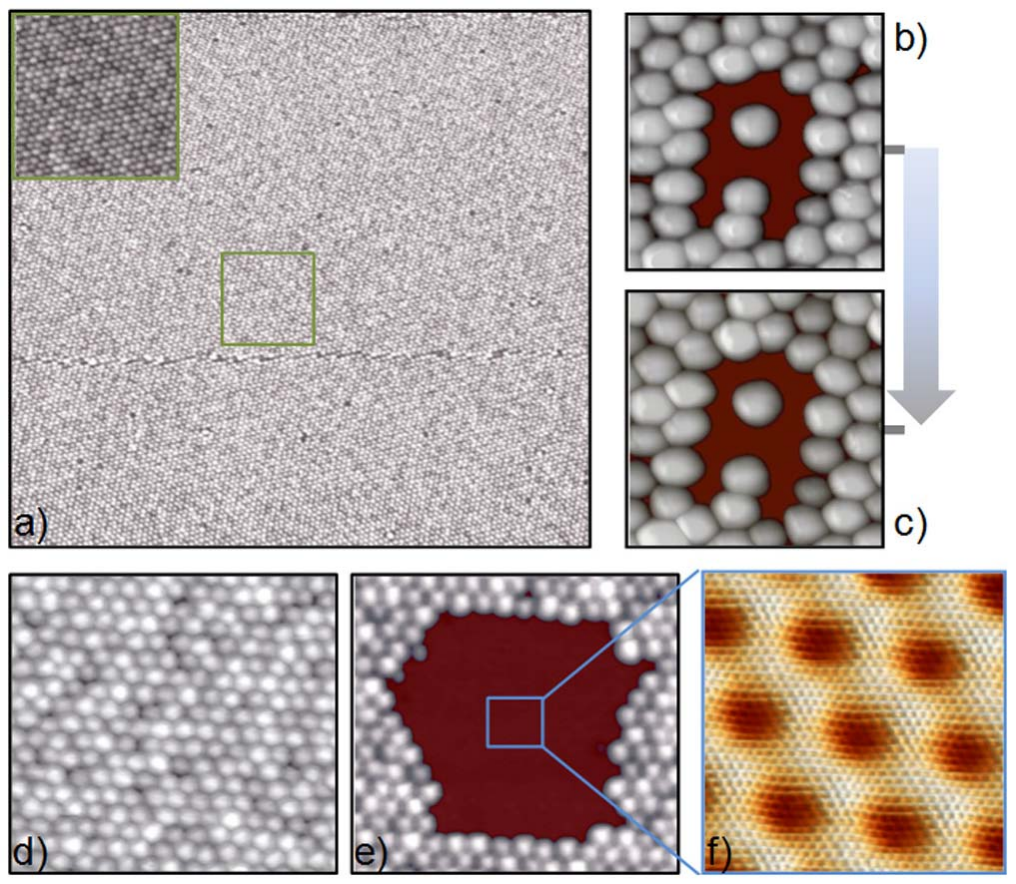
Nanostructures' size, stability and quality. (a) STM image of a 0.25 × 0.25 μm^2^ region fully covered by W clusters. Upper left inset shows a zoom of the region outlined by the green square. (b,c) 16 × 16 nm^2^ STM images of an artificially created nanostructure measured at room temperature with 24 hours difference. (d) 40 × 40 nm^2^ STM image of a W cluster superlattice on G/Ir(111). (e) STM image showing the same region as in d) after deliberately removing a large number of clusters from it. f) 7.2 × 7.2 nm^2^ STM image showing, with atomic resolution, the region outlined by a blue square in (e). Tunneling parameters: I_T_ = 50 pA, V_s_ = 1.5 V (a); I_T_ = 270 pA, V_s_ = 2.3 (b, c); I_T_ = 50 pA, V_s_ = 2.2 V(d, e); I_T_ = 5 nA, V_s_ = 35 mV (f).

Let us now address several key points to infer the actual potential of our method to architecture functional graphene nanostructures, in particular, size limits, stability and quality. The range of applicability is obviously limited by the size of the nanostructures that can be created. We can build nanostructures from the 2.5 nm limit given by the Moiré pattern distance to the few micrometers one which is given by the typical STM scanning range; the possibility of growing single Moiré patterns domains extends over several micrometers^26^. As an example, a 0.25 × 0.25 μm^2^ STM image of a graphene region uniformly covered by metallic clusters is shown in Fig 3a. Since the graphene layer grows as a carpet on top of the Ir substrate^40^, monoatomic steps, as the one existing in the middle of the image, have very little influence on the cluster superlattice. Another important question deals with the stability of the created nanostructures since any practical application would require them to be stable at room temperature. Previous studies found that the cluster superlattices as a whole are stable up to temperatures of 400 K^29^. Here, we investigated the room temperature stability of several nanostructures with very different shapes and found them to be perfectly stable within our time scale (days). As an example, we show in Figs 3b–c two STM images acquired with 24 hours difference on the same sample area where an “A” nanostructure constructed by removing 10 W clusters and presenting a single isolated cluster in its center can be appreciated. The comparison of both images clearly reveals that even complex nanostructures keep exactly the same appearance one day after their construction.

Finally, we want to comment on the state of the graphene layer after the removal of the clusters. We aim to use pristine graphene on Ir(111) domains as one of our building blocks, thus, we need our cluster extraction method to produce perfectly clean graphene regions. To this end, we show in Figs 3d–f a sequence of STM images illustrating the evolution of a region where we have removed a large number of clusters. First, we show a STM image with the pristine W cluster superlattice, see Fig. 3d. Then, in Fig 3e, we show exactly the same sample region after using the STM tip to remove all the metal clusters from its central part. Last, in Fig 3f, we show an atomically resolved STM image of the cleaned region, acquired in the central area outlined by a blue square in Fig 3e. As can be observed, no single trace of the metal clusters is found on the cleaned region which is indistinguishable from the ones obtained on pristine graphene on Ir(111) prior to the W cluster.

## Discusion

The full potential and applicability of our nanostructures is realized if the covered and uncovered regions display different electronic properties which has only been demonstrated for the homogeneous systems^2^, see Fig 1c. Scanning Tunneling Spectroscopy (STS) would seem to be the ideal tool to show if different gaps are also present in our nanostructures. Nevertheless, we were not able to obtain unambiguous data in order to detect noticeable changes in the evolution of the LDOS as the clusters were subsequently removed. In fact, for graphene on Ir(111) surfaces, dI/dV spectra seem to be mostly sensitive to a holelike surface resonance of the Ir(111) substrate rather than to any states of the graphene layer which was attributed to the selectivity of the tunneling current for states with small parallel momentum^41^. But even though transport and STS measurements are difficult due to the metallic substrate, optics and plasmonics seem within reach and in the following we discuss two new features that have the potential for sensors, metamaterials or data processing.

**Figure 4:**
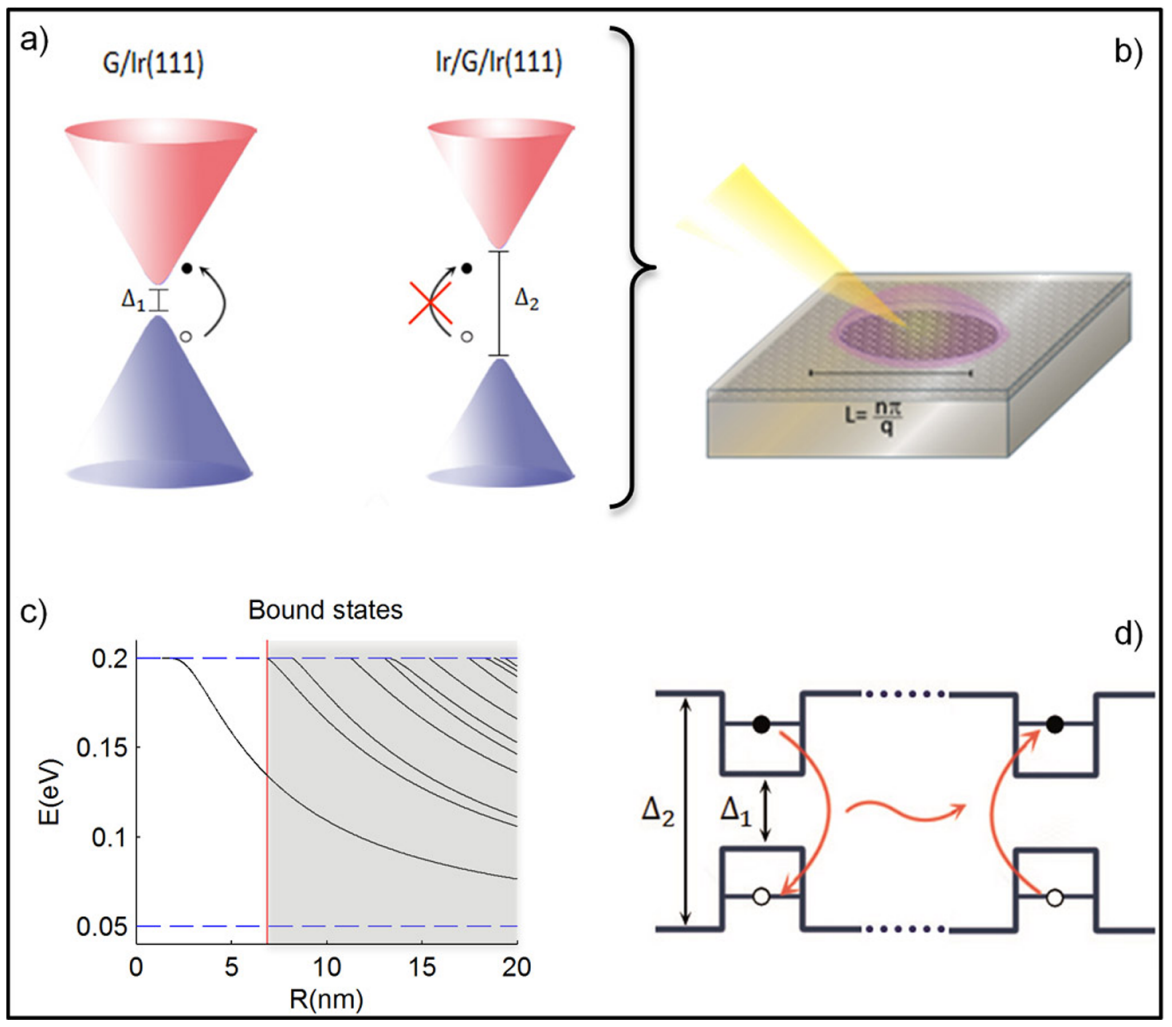
Field enhancement and mass confined quantum dots for Dirac electrons. (a) Energy dispersion near the Dirac points of the uncovered and covered areas displaying different mass gaps Δ_1_ < Δ_2_. Interband transitions with energies Δ_1_ < 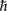
ω<Δ_2_ are assumed to be allowed in the uncovered dot, but forbidden in the surrounding region covered with Iridium clusters. (b) Schematic view of the set up leading to field enhancement by resonantly exciting interband acoustic plasmons by an electron beam or near-field techniques. (c) Energy spectrum of a circular quantum dot with radius *R* and discontinuous mass profile m = Δ_1_θ(*R – r*)+Δ_2_θ(*r – R*) where the mass parameters are Δ_1_ = 0.1 eV and Δ_2_ = 0.4 eV. The unshaded region left from the red vertical line indicates the qubit regime of only one excitonic state. (d) Schematic energy diagram of two mass confined quantum dots in the qubit regime. Electron-hole excitations (excitons) can efficiently change sites via Förster energy transfer rate which is several orders of magnitude larger than the intrinsic decay rate.

First, in the graphene/Ir(111) system, plasmonic excitations have been measured by electron energy loss spectroscopy^42^. We propose that they could be used to reach high field intensities since they are related to *π*→π* transitions between the valence and conduction band, so-called interband plasmons^43^. Assuming local band-gap variations between covered and uncovered graphene regions^2^, interband processes with transition energies 0.1 eV ≤ *E* ≤ 0.4 eV should be forbidden in the covered, but allowed in the uncovered regions and can thus be localized to small graphene areas by removing the upper Ir-clusters, see Fig. 4a. In this way, quantum dots/wires can be designed at will with 2.5 nm precision by selectively removing metallic clusters. Quantum dots/wires with diameter/width *L* posses normal modes corresponding to the wave number *q = nπ/L* of the interband plasmon (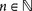), we thus expect large field enhancement due to resonant feedback effects which might be used, for instance, for spectroscopy on macro-molecules. For charge resonances at in-plane momentum *q* ≈ 0.03(0.05)Å^−1^ and energy *E* ≈ 0.25(0.375) eV^42^, the predicted field enhancement would occur for characteristic dot/wire dimensions of *L* ≈ 10.5(6.2) nm for *n* = 1 or *L* ≈ 21(12.5) nm for *n* = 2. These length scales are well within the reach of our technique (see supplementary information for details). In the same way, similar ideas can be applied to periodic structures where the excitation can be achieved also via propagating light.

A second and exciting new feature is given by the possibility to confine charged carriers, i.e., electrons as well as holes, within arbitrary geometrical regions due to locally modifying the electronic gap. One could hence design graphene quantum dots or nanowires of arbitrary size and form limited only by the cluster size of 2.5 nm which has to be contrasted with graphene nanostructures obtained by electron beam lithography and subsequent etching which have typical dimensions L = 20–100 nm. Using the effective-mass-approximation and thus the standard Dirac Hamiltonian with a variable mass profile, the discrete spectrum of a circular quantum dot as function of the radius *R* can be obtained, see Fig 4c and the supplementary information. As indicated by the unshaded region, it displays only one localized state for *R* ≤ 7 nm. In this regime, the uncovered area could resemble a quantum bit with qubit states “zero exciton” or “one exciton”. The excitonic states can further arbitrarily be connected by conventional wave function overlap or via Förster energy transfer which is mediated by the Coulomb interaction between the excitonic states, see Fig 4d. This would lead to the emergence of excitonic bands with high lifetimes as estimated via Fermi's Golden Rule (see supplementary information).

The feasibility of the above proposals crucially depends on the impact that the Iridium substrate and the clusters on top have on the electronic properties of the graphene layer. Even though from ARPES experiments the band structure hardly seems to be affected beyond the gap of Δ ≈ 0.1 eV and ≈ 0.4 eV, respectively, the graphene Dirac cone has been reported to hybridize near the Fermi level with the S1 surface state of Ir(111)^28^, and also graphene's lattice structure changes from *sp*^*2*^- to *sp*^*3*^-bonding on the covered regions^33^. Additionally, graphene optics on a metallic substrate is challenging since the induced electric dipoles in the graphene layer are usually strongly quenched by the metallic substrate. Screening effects of the underlying Iridium acting as a metallic gate will further limit the lifetime of the electron-hole pairs^47^. The implications of the Ir-substrate involving optical (q = 0) transitions, and consequently the feasibility of the proposed emergence of excitonic bands, thus need to be tested experimentally. Nevertheless, as revealed by our analysis (see SI) on the experimentally measured plasmonic dispersion on graphene on Ir(111)^42^, the screening influence of the metal on the charge excitations with finite q is surprisingly small suggesting that plasmonic excitations involving finite q-transitions should be almost unaffected by the Ir-substrate.

To conclude, we have presented a perfectly reproducible nanopatterning technique for graphene that combines bottom-up with top-down approaches. The precision is related to the periodicity of the Moiré-pattern that is formed by the graphene layer with the underlying substrate. Presupposing locally distinct electronic gaps in the covered and uncovered regions, new devices could be tailored with nano precision and we propose a novel platform for plasmonics relying on inter- rather than on intraband transitions. Also single graphene quantum dots/wires could be designed at will and arranged to arbitrary circuitries. Determining the optical gap and relaxation properties of mass-confined Dirac electrons via optical near-field scanning spectroscopy, emission spectroscopy or even transmission spectroscopy by chemically reduce the thickness of the sample would provide new insight on the role of the Ir(111) substrate on the excitonic decay rate. Finally, we note that the electronic spectrum drastically changes in the presence of a magnetic field due to the appearance of the zeroth Landau level not present in conventional semiconductor quantum dots which could be observable via Terahertz magneto-Raman spectroscopy.

## Methods

The STM experiments were performed with a home-built variable temperature instrument^44^,^45^. Tips were made of W and prepared by electrochemically etching and subsequently annealing in UHV conditions. STM data were acquired with a fully automated workstation that incorporates digital feedback control based on DSP (digital signal processor) technology. All the surface manipulation experiments, data acquisition, and image processing were performed using the WSxM software^46^. STM images were all acquired in the constant current mode.

### Sample preparation

Ir(111) surfaces were cleaned by 1 keV Ar^+^ sputtering at 850°C. The growth of graphene on the clean Ir(111) surface was performed by chemical vapor deposition (CVD) of ethylene (3 × 10*^−^*^7^ Torr during 1 min) in UHV environments with the Ir(111) substrate held at 1050°C. Under such conditions, small areas of the Ir(111) substrate remained intentionally uncovered by graphene, which allowed us to estimate the coverage of W or Ir used for the cluster formation. W and Ir were evaporated from high purity filaments composed of each corresponding material. An accurate calibration of the deposition rate as a function of the filament temperature, measured by an infrared pyrometer, was performed by means of STM images acquired on areas of bare -uncovered by graphene- Ir(111).

### Theory and Modeling

The experimental data (plasmonic excitations in graphene on Ir(111) and Pt(111)) was obtained from the original publications and fitted to the theoretical predictions using the least-square method. The electronic properties of graphene were modeled using the standard effective-mass-approximation. Exciton lifetimes and hopping amplitudes were estimated via Fermi's Golden Rule.

## Author Contributions

A.J.M.G. carried out the experiments supported by I.B. and J.M.G.R. A.J.M.G. and J.M.G.R. designed the research helped by I.B. A.J.M.G., I.B and J.M.G.R. analyzed the data. T.S. performed the main calculations with the collaboration of A.G.R. I.B. wrote the manuscript with the help of T.S. All authors contributed to the scientific discussion and revised the manuscript.

## Supplementary Material

Supplementary Information Supplementary Material for the article Towards scalable nano-engineering of graphene

Supplementary Information Nano-lithographing Graphene with 2.5nm precision

Supplementary Information Nano-lithographing Ir on graphene with 2.5nm precision

Supplementary Information Nano-lithographing W on graphene with 2.5nm precision
