# Supplementary material for: Towards scalable nano-engineering of graphene
Source: Sci Rep. 2014 Dec 4;4:7314. doi: 10.1038/srep07314 (PMC4255185; doi:10.1038/srep07314)
Supplement: Supplementary Information — Supplementary Material for the article Towards scalable nano-engineering of graphene [file srep07314-s1.pdf]

Supplementary Material for the article

"Towards scalable nano-engineering of graphene"

A.J. Martínez-Galera[1], I. Brihuega[1,2,*], A. Gutiérrez-Rubio[1,3],
T. Stauber[1,2,3], and J. M. Gómez-Rodríguez[1,2]

[1] *Departamento de Física de la Materia Condensada,*
Universidad Autónoma de Madrid, E-28049 Madrid, Spain

[2] *Departamento de Física de la Materia Condensada,*
Universidad Autónoma de Madrid, E-28049 Madrid, Spain

[3] *Instituto de Ciencia de Materiales de Madrid,*
Consejo Superior de Investigaciones Cientficas, E-28049 Madrid, Spain

**) Corresponding author: ivan.brihuega@uam.es*

EXPERIMENTAL DETAILS

Sample preparation

The experiments were carried out in an ultra-high-vacuum (UHV) system whose base pressure is below 5×10^{-11} Torr. The system is equipped with a variable temperature scanning tunneling microscope (VT-STM), low energy electron diffraction (LEED), Auger electron spectroscopy (AES), sample and STM tip transfer and heating capabilities, an STM tip cleaning system by field emission, several interchangeable evaporation cells, a quartz crystal microbalance, and an ion gun for sample cleaning purposes.

The STM experiments were performed with a home-built variable temperature instrument. Tips were made of W and prepared by electrochemically etching and subsequently annealing in UHV conditions. STM data were acquired with a fully automated workstation that incorporates digital feedback control based on DSP (digital signal processor) technology[1]. All the surface manipulation experiments, data acquisition, and image processing were performed using the WSxM software[2]. STM images were all acquired in the constant current mode.

Ir(111) surfaces were cleaned by 1 keV Ar^+ sputtering at 850 °C. The growth of graphene on the clean Ir(111) surface was performed by chemical vapor deposition (CVD) of ethylene (3×10^{-7} Torr during 1 min) in UHV environments with the Ir(111) substrate held at 1050 °C. Under such conditions, small areas of the Ir(111) substrate remained intentionally uncovered by graphene, which allowed us to estimate the coverage of W or Ir used for the cluster formation (see below). W and Ir were evaporated from high purity filaments composed of each corresponding material. An accurate calibration of the deposition rate as a function of the filament temperature, measured by an infrared pyrometer, was performed by means of STM images acquired on areas of bare -uncovered by graphene- Ir(111). Figure S1 shows a representative STM topograph acquired after the adsorption of 0.50 ± 0.05 ML of W at a deposition rate of 8×10^{-3} ML/s on a graphene/Ir(111) surface at room temperature. Here, 1 ML corresponds to the atomic density of the Ir(111) surface. As can be appreciated in Fig. S1, a W cluster superlattice with high structural perfection extending over very large areas of the graphene/Ir(111) surface is formed.

Extraction probability

We acquired more than 1000 curves in order to obtain the extraction probability shown in Fig. 2l of the main manuscript. Each single point in this graph corresponds to the probability of removing a W cluster by approaching the STM tip towards the sample a certain distance with a given applied sample voltage. For each point, this probability was obtained by performing 20 single attempts to extract 20 different clusters. In each attempt, we set the stabilization current to 70 pA at the selected sample voltage and then, with the selected voltage fixed, we approached the tip towards the surface the chosen distance at a constant rate. After each single attempt, we measured a STM image to check whether the selected cluster was removed or not. The extraction probability for each point of the graph corresponds then to the total number of clusters removed in these 20 attempts divided by 20. Error bars correspond to the standard deviation of a binomial distribution.

Conductance histogram

We constructed the conductance histogram shown in Fig. 2m of the main manuscript by recording the current during the vertical displacement of the STM tip (I-Z curves) on 1200 single W cluster extraction events. Each I-Z curve was obtained, at RT, by approaching 1.0 nm the STM tip towards the sample, with 0.1 V sample voltage and stabilization current of 70 pA before opening the feedback loop. In all cases we verified by STM that the metallic cluster was successfully removed. Such conductance histogram was required to extract some meaningful information from the experimental data since, as explained in the main text, individual conductance curves so acquired were inherently irreproducible. In Fig. S2, we present four typical I-Z curves, each of them acquired during the extraction of a W cluster, to illustrate this point.

THEORETICAL DETAILS

Acoustic interband plasmons in graphene on Iridium(111) and field enhancement

In this section, we will propose that experimentally observed plasmonic resonances in graphene on Ir(111) [T. Langer et al., New J. Phys. **13***, 053006 (2011)] are due to interband transitions and that the screening influence of the metal on the charge excitations with finite wave number is surprisingly small.*

In Ref. 3, graphene on Iridium(111) was investigated by means of high resolution electron energy-loss spectroscopy (EELS). A characteristic peak in the loss function with linear dispersion from energies $\hbar\omega = 0.1$eV up to $\hbar\omega = 1.5$eV was seen. Additionally, the width of the resonance showed linear behavior. Both features can be captured by a simple theory assuming the conductivity for undoped graphene including only interband transitions,[4]

$$\sigma(q,\omega) = \sigma_0 \frac{\omega}{\sqrt{\omega^2 - (v_F q)^2}} \;, \tag{1}$$

with $\sigma_0 = e^2/(4\hbar)$ the universal conductivity and the Fermi velocity v_F. With the dielectric function

$$\epsilon(q,\omega) = 1 + \frac{\mathrm{i}\sigma(q,\omega)q}{2\omega\epsilon\varepsilon_0} \;, \tag{2}$$

and the (generally complex) static dielectric constant $\epsilon = \epsilon' + \mathrm{i}\epsilon''$, the energy loss function $S = -\mathrm{Im}\,\epsilon^{-1}$ shows a maximum at $x = 1$ with

$$x = \frac{\pi\alpha_g}{2|\epsilon|}\frac{v_F q}{\sqrt{\omega^2 - (v_F q)^2}} \;, \tag{3}$$

$|\epsilon| = \sqrt{\epsilon'^2 + \epsilon''^2}$ and graphene's fine-structure constant $\alpha_g = \alpha\frac{c}{v_F} \approx 2.2$ where $\alpha = e^2/(4\pi\varepsilon_0\hbar c) \approx 1/137$. This corresponds to charge excitations with a linear dispersion $\omega = v_s q$ where the sound-velocity reads $v_s = \sqrt{1 + \left(\frac{\pi\alpha_g}{2|\epsilon|}\right)^2} v_F$. For $|\epsilon| \approx 3.5$, we obtain a good fit to the experimentally observed sound velocity of $v_s \approx 1.4 v_F$ for Iridium.[3]

EELS experiments were also performed for graphene on SiC[5] and on Platinum.[6] Again, a characteristic peak in the loss function with linear dispersion was observed with sound velocities $v_s \approx 1.4 v_F$ for a SiC-substrate and $v_s \approx 1.15 v_F$ for a Platinum substrate. Applying the same theory as above, i.e., only including interband transitions, we obtain good fits to

the experimental data for $|\epsilon| \approx 3.5$ (SiC) and $|\epsilon| \approx 6.1$ (Platinum), respectively. For a dielectric substrate, the dielectric response is real, $|\epsilon| \approx \epsilon'$, leading to the usual dielectric constant for SiC $\epsilon_{SiC} \approx 6$, where we used $\epsilon' = (\epsilon_{SiC}+1)/2$ valid for a substrate/graphene/air interface. This confirms that assuming interband plasmonic resonances leads to consistent results.

We can refine the above approach and also include the band gap Δ that appears in the spectrum of graphene on Iridium(111). For that, we need to consider the conductivity of gapped graphene which also acquires an imaginary part. The longitudinal part that couples to charge fluctuations reads[7]

$$\mathrm{Re}\left\{\sigma(q,\omega)\right\} = \sigma_0 \sqrt{1-\left(\frac{v_F q}{\omega}\right)^2}^{\,-1} \left[1+\frac{\Delta^2}{\hbar^2(\omega^2-(v_F q)^2)}\right] \theta\left((\hbar\omega)^2-(\hbar v_F q)^2-\Delta^2\right); \tag{4}$$

$$\mathrm{Im}\left\{\sigma(q,\omega)\right\} = \frac{\sigma_0}{\pi}\left\{\frac{2\Delta}{\hbar\omega}\frac{\omega^2}{\omega^2-(v_F q)^2} + \frac{2\omega}{\sqrt{|(v_F q)^2-\omega^2|}}\left[1+\frac{\Delta^2}{\hbar^2(\omega^2-(v_F q)^2)}\right]\times\right. \tag{5}$$

$$\left.\times\left[\theta(v_F q-\omega)\arccos\left(\frac{\Delta}{\sqrt{\Delta^2+\hbar^2((v_F q)^2-\omega^2)}}\right) - \theta(\omega - v_F q)\frac{1}{2}\log\left|\frac{\hbar\sqrt{\omega^2-(v_F q)^2}+\Delta}{\hbar\sqrt{\omega^2-(v_F q)^2}-\Delta}\right|\right]\right\}.$$

On the left hand side of Fig. S3, the electron loss function $S = -\mathrm{Im}\,\epsilon^{-1}$ with the conductivity of gapped graphene, Eqs. (4) and (5), is shown. We also plot the experimental data of Ref. 3 (squares) and the linear dispersion $\omega = v_s q$ with sound velocity $v_s = \sqrt{1+\left(\frac{\pi\alpha_g}{2|\epsilon|}\right)^2} v_F$ for $|\epsilon| \approx 3.5$ (solid line). There are no significant changes compared to the theory of ungapped graphene. This suggests that the charge resonances seen in typical EELS experiments are due to interband transitions which can be confined up to a certain energy by a larger gap present in the surrounding region. An analogous analysis for gapless but doped graphene on Pt is carried out on the right hand side of Fig. S3, again yielding a good fit for $|\epsilon| \approx 6.1$. Let us finally note that both dielectric constants for Iridium as well as for Platinum are considerably lower than expected for a typical metal which suggests that optics for frequencies ranging from THz to mid-infrared associated with finite wave numbers is possible even in the presence of a metallic substrate.

Spectrum of mass-confined graphene quantum dots

In this section, we discuss the spectrum of spherical graphene quantum dots. Electronic localization is obtained due to a different mass-profile presumably provoked by the presence or absence of the metallic nanoclusters.

The removal of Ir-clusters on top of graphene on Iridium(111) presumably creates Dirac quantum dots with nanoscale dimensions due to mass confinement. Dirac carriers localized by a variable mass profile lead to well-defined discrete states with high intrinsic lifetime due to the absence of typical edge disorder. We model the confined region by a circular quantum dot of radius R where a step-like change in the mass term $2mv_F^2 = \Delta_1\theta(R-r)+\Delta_2\theta(r-R)$ with $\Delta_1 = 0.05$eV for the dot and $\Delta_2 = 0.4$eV for the bulk region. The Hamiltonian of a gapped graphene sheet in polar coordinates reads

$$\hat{H} = \begin{bmatrix} mv_F^2 & -i\hbar v_F e^{-i\theta}\left[\partial_r - \frac{i}{r}\partial_\theta\right] \\ -i\hbar v_F e^{i\theta}\left[\partial_r + \frac{i}{r}\partial_\theta\right] & -mv_F^2 \end{bmatrix} . \tag{6}$$

With $l \in \mathbb{Z}$, the eigenfunctions can be written in the general form

$$\psi_{E,l}(\vec{r}) = \langle \vec{r}|El\rangle = \begin{bmatrix} f_{E,l}(r)e^{il\theta} \\ g_{E,l}(r)e^{i(l+1)\theta} \end{bmatrix} . \tag{7}$$

For the given mass profile and $\Delta_1 < 2|E| < \Delta_2$, we explicitly have

$$f_{E,l}(r) = \begin{cases} Bj_l(qr) & \text{for } r < R \\ BA_l h_l^{(1)}(kr) & \text{for } r > R \end{cases} , \tag{8}$$

$$g_{E,l}(r) = \begin{cases} Bi\eta_q j_{l+1}(qr) & \text{for } r < R \\ BA_l i\eta_k h_{l+1}^{(1)}(kr) & \text{for } r > R \end{cases} , \tag{9}$$

with j_l the Bessel and $h_l^{(1)} = j_l + \mathrm{i}y_l$ the Hankel function where y_l denotes the Neumann function. We further have with $\Delta_1 = 2m_1v_F^2$ and $\Delta_2 = 2m_2v_F^2$

$$A_l = \frac{j_l(qR)}{h_l^{(1)}(kR)} , \tag{10}$$

$$q = \sqrt{(E - m_1v_F^2)(E + m_1v_F^2)}/\hbar v_F , \qquad \eta_q = \mathrm{sg}(E)\sqrt{\left|\frac{E - m_1v_F^2}{E + m_1v_F^2}\right|} , \tag{11}$$

$$k = i\sqrt{|(E - m_2v_F^2)(E + m_2v_F^2)|}/\hbar v_F , \qquad \eta_k = i\sqrt{\left|\frac{E - m_2v_F^2}{E + m_2v_F^2}\right|} , \tag{12}$$

and B is a normalization constant. The matching conditions on the frontier R yield the

following equation:

$$\eta_k h^{(1)}_{l+1}(kR) j_l(qR) = \eta_q j_{l+1}(qR) h^{(1)}_l(kR) \; . \tag{13}$$

Its solutions are given by the discrete bound state energies. These are shown in Fig. S4, where the black curves correspond to $l \geq 0$ and the red curves to $l < 0$. On the left hand side of Fig. S5, the eigenfunctions of Eq. (8) and (9) are plotted for the first three electronic energy levels of a dot with radius $R = 15$nm and on the right hand side the probability density $|\psi_{E,l}(\vec{r})|^2$.

There is a bound state at $E \approx 0.2$eV for a radial quantum dot even with radius $R = 2.5$nm. This energy is lowered by excitonic effects and also due to a smooth effective mass evolution which tends to decrease the electron confinement. Changes might also occur due to strain effects leading to effective magnetic fields.[8] We thus predict a bound exciton even in small quantum dots with radius $R = 2.5$nm to be verified by photoluminescence or near-field spectroscopy. For larger, not necessarily circular quantum dots, we also expect at least one bound state in the conduction as well as in the valence band, see Fig. S4. For quantum dots with more than one bound state, the relaxation mechanisms to the ground state due to various mechanisms such as optical/acoustic phonons or Auger electrons can be discussed in detail as the dot size can be increased till the two-dimensional limit has been reached.

Excitonic bands in arbitrary quantum dot arrays

In this section, we will discuss possible extensions and future research directions that our approach offers. For explicit calculations, we assume a non-dissipative substrate which needs to be improved in subsequent calculations after the screening influence of the metal has been experimentally investigated.

Graphene optics on a metallic substrate is challenging since the induced electric dipoles in the graphene layer are usually strongly quenched by the metallic substrate. Nevertheless, the previous analysis on the acoustic plasmonic excitations showed that the influence for finite q-numbers is considerably less than expected, especially for Iridium, leading to a static dielectric constant $|\epsilon| \approx 3.5$. This means that screening due to induced particle-hole processes in Iridium is suppressed and that the lifetime of graphene's electron-hole excitations is only weakly limited by the substrate. The suppressed fluorescent quenching in the relevant (optical) regime would open up the possibility for optical experiments on

graphene/Ir(111) at THz to mid/near infrared frequencies. This holds even more if finite wave numbers q are involved in the absorption process which can be the case in periodic superstructures.

In the following, we will thus neglect intrinsic damping and assume a *real* dielectric constant ϵ. Then, only the spontaneous decay of the excitons must be considered when evaluating their lifetimes. This decay rate is given by[9]

$$\gamma = \frac{\omega^3 |\vec{\mu}|^2}{3\pi\varepsilon_0 \epsilon \hbar c^3}, \tag{14}$$

where ω and $\vec{\mu}$ are the energy and the dipole moment of the exciton, respectively, ε_0 the vacuum permittivity and ϵ the real (average) dielectric constant, i.e., $\epsilon = (\epsilon_{substrate} + 1)/2$ for a substrate/graphene/air interface. For graphene, we can write this as

$$\gamma = \frac{4\alpha\omega}{3\epsilon} \left(\frac{v_F}{c}\right)^2 |\langle El|\sigma_x|E'l'\rangle|^2 \tag{15}$$

with $|El\rangle$ ($|E'l'\rangle$) the electron (hole) bound state, $\hbar\omega = E - E'$, α the fine-structure and σ_x the x-component of the Pauli-matrices. In a setup with only one bound state in the valence and conduction band, like the one depicted in Fig. S6, one has $E = -E' = \hbar\omega/2$ and $l = 0$, $l' = -1$. For $R = 5$nm and $\epsilon \approx 3.5$, this yields $\gamma \simeq 2 \cdot 10^7 \mathrm{s}^{-1}$ at transition energy $\hbar\omega \approx 0.3$eV.

Let us now assume a quantum dot in the first excited (excitonic) state next to a second quantum dot at distance D of the same dimension that is in its ground state. Since they have the same excitation spectrum there is the possibility for efficient energy (Förster) transfer from one dot to the other, see process 2 in Fig. S6. If this non-radiative process is considerably faster than the spontaneous decay rate of the exciton, a hopping mechanism for the exciton is induced and arbitrary two-dimensional lattices could be designed with the life-time limited only by intrinsic dissipative effects like phonon and bulk-electron scattering.

The transition probability t_n of an exciton hopping from one quantum dot to another mediated by the inter-dot Coulomb interaction V_C shall be estimated using Fermi's Golden rule,

$$t_n = \frac{2\pi}{\hbar} \sum_m |\langle n|V_C|m\rangle|^2 \delta(\epsilon_n - \epsilon_m) \ , \tag{16}$$

where $|m\rangle$ and $|n\rangle$ are excitonic states. The delta function is usually replaced by the absorption and emission spectra of the donor and acceptor. We will thus assume homogeneously broadened excitonic states $\rho_n(\epsilon) = \pi^{-1}\Gamma/((\epsilon - \epsilon_n)^2 + \Gamma^2)$ and replace the δ-function by $\delta_{n,m} = \int d\epsilon \rho_n(\epsilon)\rho_m(\epsilon)$.[10] Conventional overlap of the wave functions of quantum dots leads to an additional hopping mechanism.

We model the excitonic state as product state of the electron and hole wave function. The matrix element is thus given by $\langle El\vec{r}_2; E'l'\vec{r}_1|V_C|El\vec{r}_1; E'l'\vec{r}_2\rangle$, where $E' < 0$, $E > 0$ and V_C the Coulomb interaction with

$$\langle \vec{r}_3 s_3; \vec{r}_4 s_4|V_C|\vec{r}_1 s_1; \vec{r}_2 s_2\rangle = \frac{e^2}{4\pi\varepsilon_0\epsilon|\vec{r}_1 - \vec{r}_2|}\delta_{s_1,s_3}\delta_{s_2,s_4}\delta(\vec{r}_1 - \vec{r}_3)\delta(\vec{r}_2 - \vec{r}_4)\ . \tag{17}$$

The two (direct and exchange) processes are depicted in Fig. S6.

Let us consider the special case of only one excitonic bound state, i.e., quantum dots with radius $R \lesssim 10$nm and we drop the subindex $t_n \to t$. For typical broading $\Gamma = 10$meV and dot radius $R = 5$nm, the hopping probabilities for the two non-radiative processes, depicted in Fig. S6, as well as for conventional wave function overlap are plotted in Fig. S7 as a function of the inter-dot distance D.

The Förster energy transfer is dominant for $D > 6R$ displaying the typical algebraic decay $t \sim D^{-6}$ obtained from a multipolar expansion. In this regime, the excitonic hopping induced by this mechanism is two orders of magnitude greater than the spontaneous decay rate γ. As a consequence, a well-defined band of Frenkel excitons is expected with Wannier functions well approximated by the individual quantum dot wave functions. On the other hand, for $D < 6R$, Wannier functions describing the exciton will be more spread and the resultant excitons would be more delocalized. A different approach including the array of quantum dots from the beginning is then necessary.

To conclude, our results demonstrate that depending on the dimensions of the quantum dots and the distance between them, we can engineer lattices hosting different kinds of excitons (Frenkel vs. Wannier) and with tunable excitonic hoppings between the dots. This would provide an alternative platform to study the dynamics in quantum mechanical tight-binding models and also the possibility of discussing exciton-polariton condensation[11] in a controllable system.

Acknowledgments

We thank F. Guinea, G. Gómez-Santos and B. Amorim for helpful discussions. This work has been supported by FCT under grants PTDC/FIS/101434/2008; PTDC/FIS/113199/2009 and MIC under grant FIS2010-21883-C02-02.

---

[1] Nanotec Electrónica S.L., http//www.nanotec.es.

[2] I. Horcas, R. Fernández, J. M. Gómez-Rodríguez, J. Colchero, J. Gómez-Herrero, and A. M. Baro, Review of Scientific Instruments **78**, 013705 (2007).

[3] T. Langer, D. F. Förster, C. Busse, T. Michely, H. Pfnür, and C. Tegenkamp, New Journal of Physics **13**, 053006 (2011).

[4] T. Stauber, Journal of Physics: Condensed Matter **26**, 123201 (2014).

[5] Y. Liu, R. F. Willis, K. V. Emtsev, and T. Seyller, Phys. Rev. B **78**, 201403 (2008).

[6] A. Politano, A. R. Marino, V. Formoso, D. Farías, R. Miranda, and G. Chiarello, Phys. Rev. B **84**, 033401 (2011).

[7] A. Scholz and J. Schliemann, Phys. Rev. B **83**, 235409 (2011).

[8] F. Guinea, M. I. Katsnelson, and A. K. Geim, Nature Phys. **6**, 30 (2010).

[9] L. Novotny and B. Hecht, *Principles of Nano-Optics* (Cambridge University Press, Cambridge, 2006).

[10] A. O. Govorov, Phys. Rev. B **68**, 075315 (2003).

[11] A. Amo, D. Sanvitto, F. P. Laussy, D. Ballarini, E. d. Valle, M. D. Martin, A. Lemaitre, J. Bloch, D. N. Krizhanovskii, M. S. Skolnick, C. Tejedor, and L. Vina, Nature **457**, 291 (2007).

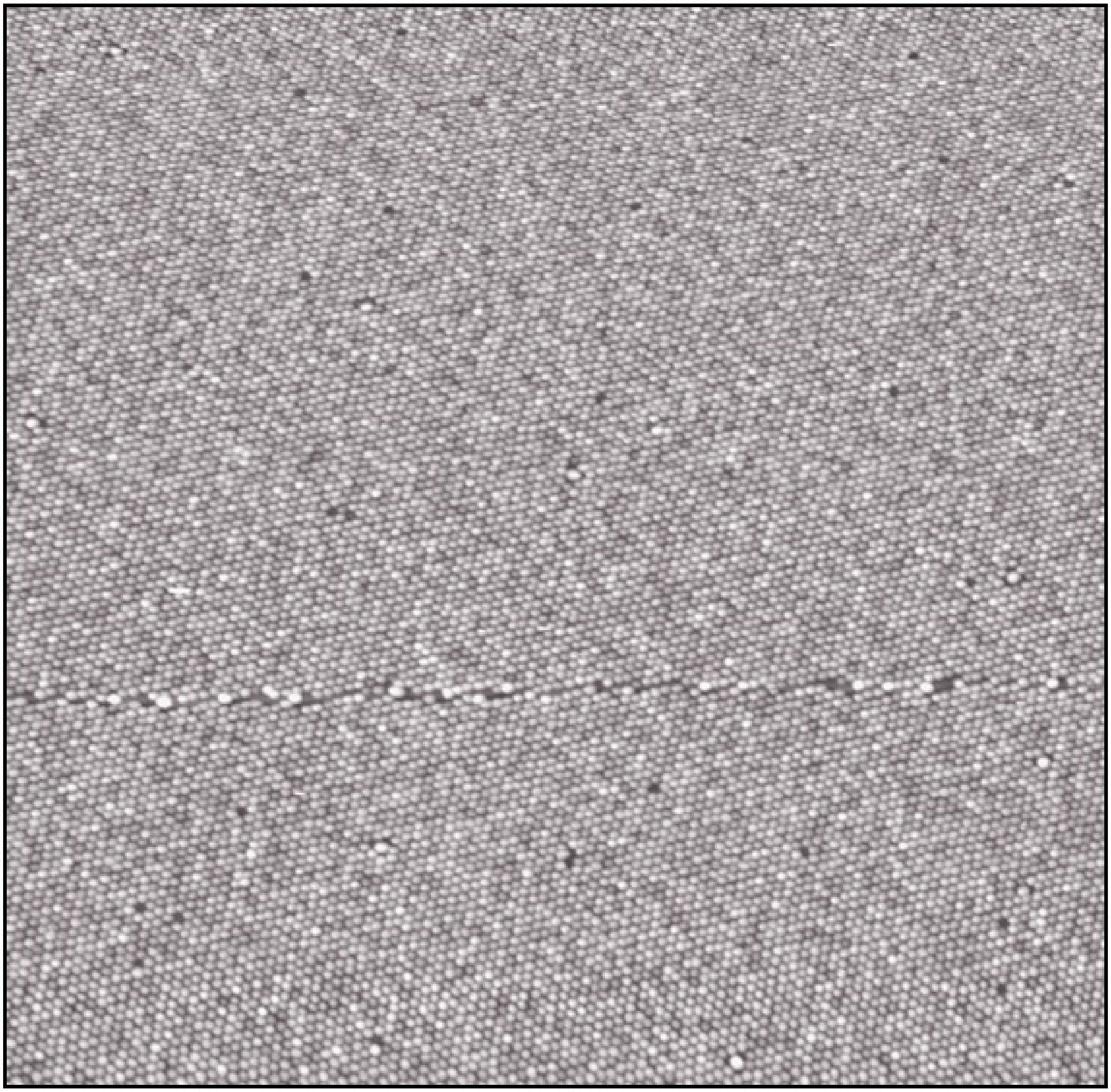

Fig. S1: Sample morphology. STM image of a $0.25 \times 0.25\,\mu\mathrm{m}^2$ region fully covered by W clusters. Sample voltage: $1.5\,\mathrm{V}$; tunneling current: $50\,\mathrm{pA}$.

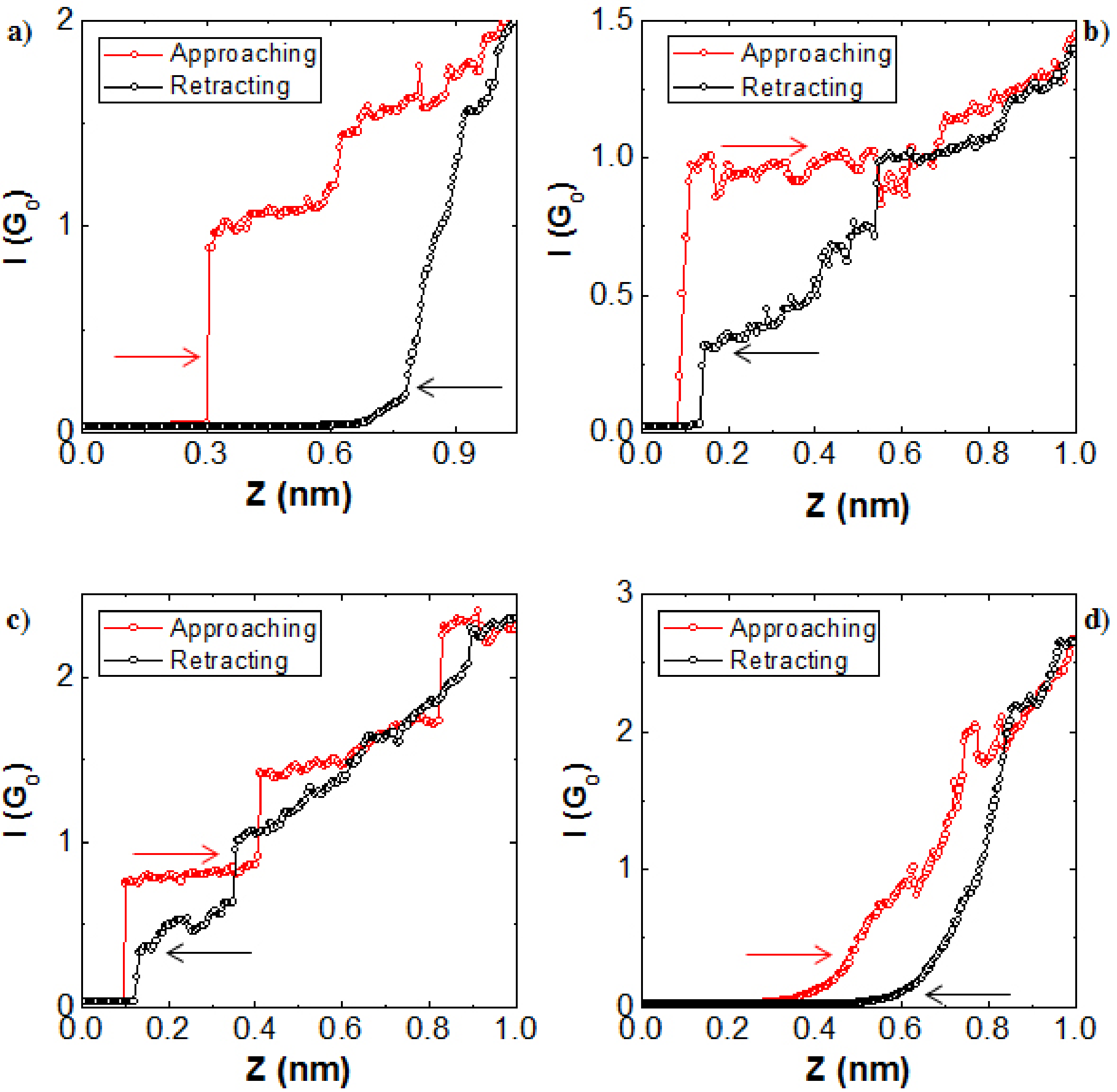

Fig. S2: Characteristic I-Z curves illustrating the different behavior of the conductance traces in the cluster extraction process. All the curves were measured at RT with the same tunneling conditions; sample voltage = +100 mV, stabilization current = 70 pA.

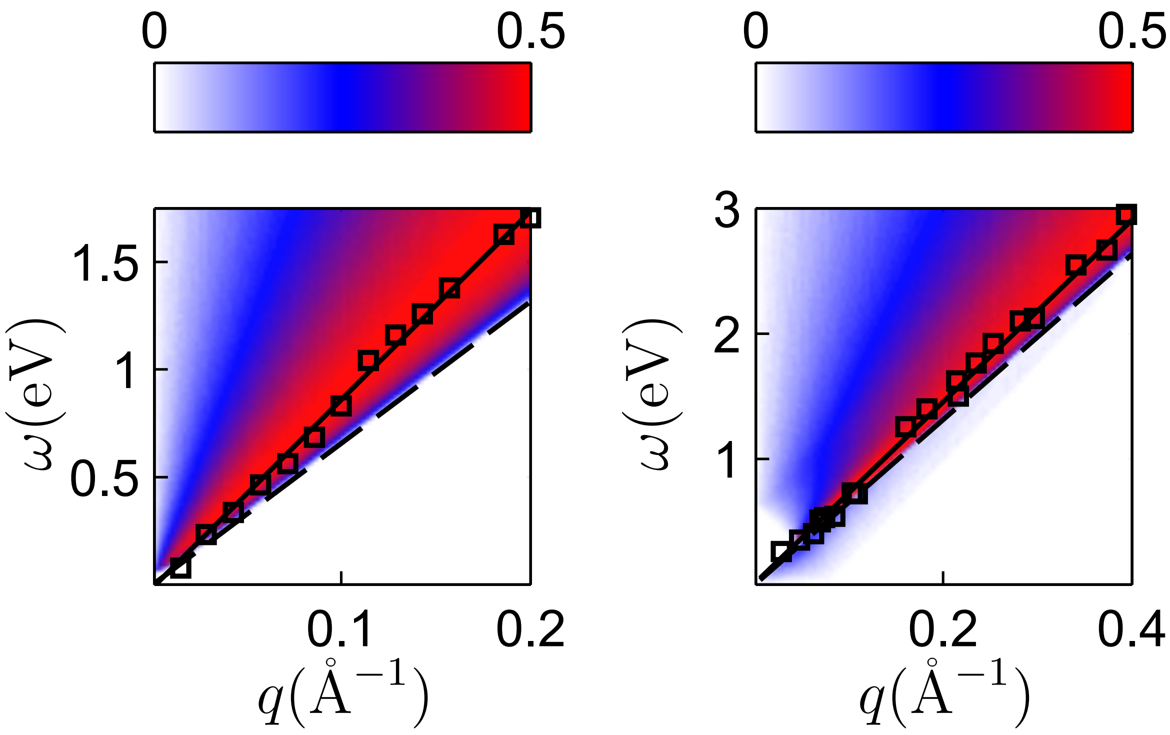

Fig. S3: Loss function of graphene on top of an Iridium (left) and Platinum (right) substrate due to interband transitions compared to the experimental data of Ref. 3 and 6 (squares), respectively. Also shown the acoustic plasmon dispersion $\omega = v_s q$ with sound velocity $v_s \approx \sqrt{1 + (\frac{\pi\alpha_g}{2|\epsilon|})^2} v_F$ for dielectric constants $|\epsilon| = 3.5$ (left) and $|\epsilon| = 6.1$ (right). The dashed line indicates the Dirac dispersion $\omega = v_F q$.

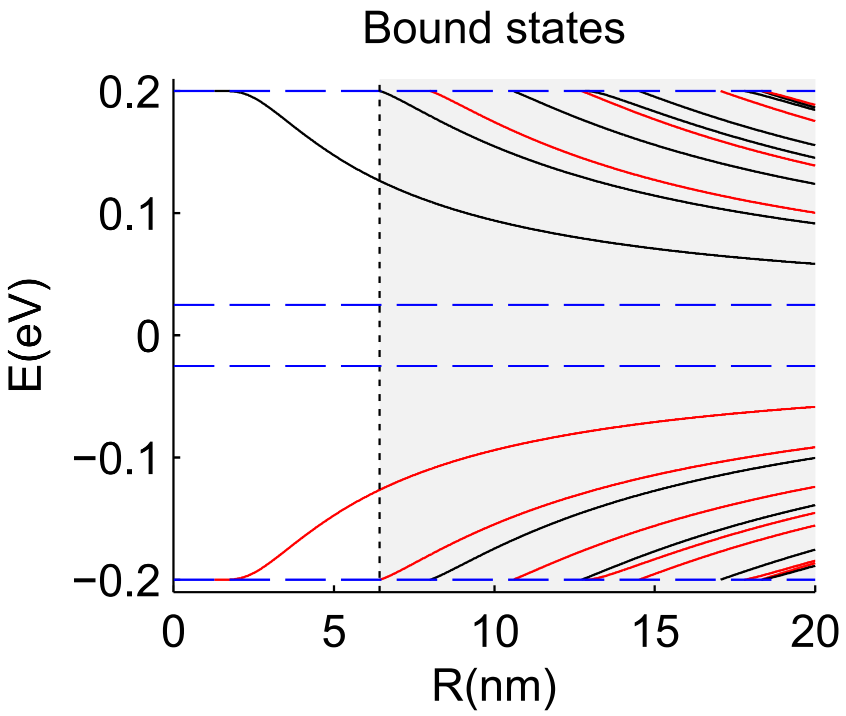

Fig. S4: Band structure of a circular graphene quantum dot with step-like mass profile $2mv_F^2 = \Delta_1\theta(R-r) + \Delta_2\theta(r-R)$ with $\Delta_1 = 0.05$eV for the dot and $\Delta_2 = 0.4$eV for the bulk region. Black curves correspond to $l \geq 0$ and red curves to $l < 0$. To every curve with l corresponds a particle hole symmetric curve with $-l-1$.

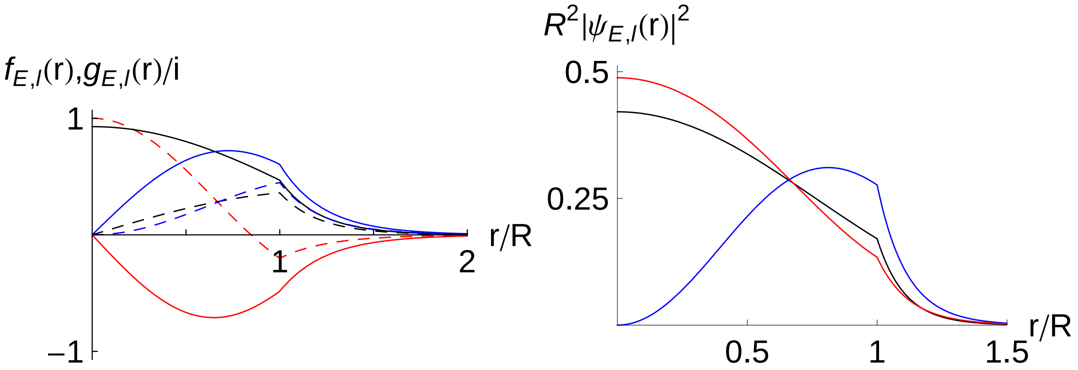

Fig. S5: Left hand side: $f_{E,l}(r)$ (solid lines) and $g_{E,l}(r)/\mathrm{i}$ (dashed lines) in arbitrary units for a quantum dot with radius $R = 15\,\mathrm{nm}$. The three lowest bound states with positive energy are shown in increasing order as black (lowest energy), blue and red curves which correspond to $l = 0$, $l = 1$ and $l = -1$, respectively. Right hand side: Probability density of the aforementioned bound states with $|\psi_{E,l}(r)|^2 = |f_{E,l}(r)|^2 + |g_{E,l}(r)|^2$.

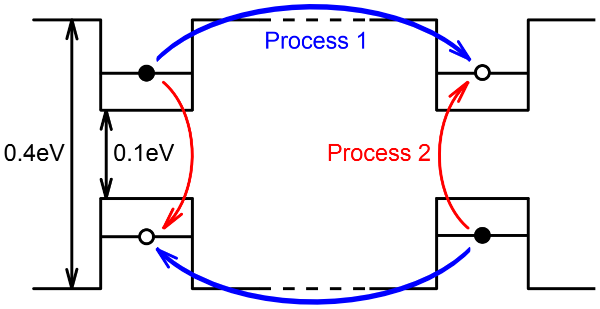

Fig. S6: Schematic band structure of the graphene quantum dot localized by a mass-term and the energy transfer from one dot to another via different processes mediated by the Coulomb interaction. Förster energy transfer corresponds to process 2.

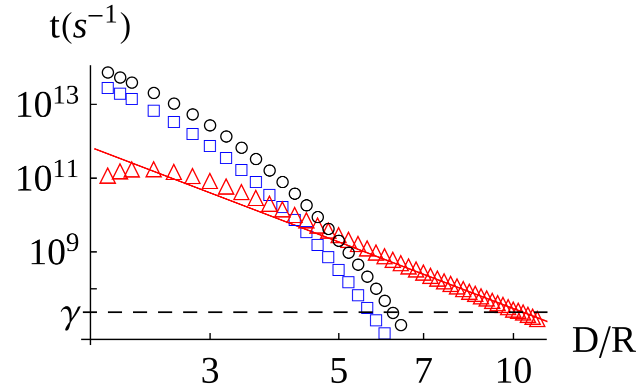

Fig. S7: (color online): Transition rates of different hopping processes between two quantum dots as function of the distance D (see Fig. S6). Blue squares: process 1; red triangles: process 2; black circles: wave functions overlap. The solid red line corresponds to the multipolar expansion of the Coulomb potential and shows the typical algebraic Förster decay $t \sim D^{-6}$.
